# The p38/MK2/Hsp25 Pathway Is Required for BMP-2-Induced Cell Migration

**DOI:** 10.1371/journal.pone.0016477

**Published:** 2011-01-28

**Authors:** Cristina Gamell, Antonio G. Susperregui, Ora Bernard, José Luis Rosa, Francesc Ventura

**Affiliations:** 1 Departament de Ciències Fisiològiques II, Universitat de Barcelona, IDIBELL, L'Hospitalet de Llobregat, Spain; 2 St. Vincent's Institute of Medical Research, Victoria, Australia; Instituto de Medicina Molecular, Portugal

## Abstract

**Background:**

Bone morphogenetic proteins (BMPs) have been shown to participate in the patterning and specification of several tissues and organs during development and to regulate cell growth, differentiation and migration in different cell types. BMP-mediated cell migration requires activation of the small GTPase Cdc42 and LIMK1 activities. In our earlier report we showed that activation of LIMK1 also requires the activation of PAKs through Cdc42 and PI3K. However, the requirement of additional signaling is not clearly known.

**Methodology/Principal Findings:**

Activation of p38 MAPK has been shown to be relevant for a number of BMP-2′s physiological effects. We report here that BMP-2 regulation of cell migration and actin cytoskeleton remodelling are dependent on p38 activity. BMP-2 treatment of mesenchymal cells results in activation of the p38/MK2/Hsp25 signaling pathway downstream from the BMP receptors. Moreover, chemical inhibition of p38 signaling or genetic ablation of either p38α or MK2 blocks the ability to activate the downstream effectors of the pathway and abolishes BMP-2-induction of cell migration. These signaling effects on p38/MK2/Hsp25 do not require the activity of either Cdc42 or PAK, whereas p38/MK2 activities do not significantly modify the BMP-2-dependent activation of LIMK1, measured by either kinase activity or with an antibody raised against phospho-threonine 508 at its activation loop. Finally, phosphorylated Hsp25 colocalizes with the BMP receptor complexes in lamellipodia and overexpression of a phosphorylation mutant form of Hsp25 is able to abolish the migration of cells in response to BMP-2.

**Conclusions:**

These results indicate that Cdc42/PAK/LIMK1 and p38/MK2/Hsp25 pathways, acting in parallel and modulating specific actin regulatory proteins, play a critical role in integrating responses during BMP-induced actin reorganization and cell migration.

## Introduction

Cell migration is essential for important biological processes such as embryonic morphogenesis, wound healing, inflammatory responses, angiogenesis or tumor metastasis. It involves spatially and temporally coordinated events: formation of actin-rich protrusions such as lamellipodia, their adhesion, translocation of the cell body and rear detachment [1]. Various proteins participate in the modulation of actin cytoskeleton reorganization in response to migration promoting agents. Actin filaments at the leading edge of lamellipodia are organized as a branched network which is polarized, with barbed ends oriented towards the membrane [1], [2].

Critical players in this process are the Arp2/3 complex and its activators WASP/Scar which transduce the activating signals emanating from the Rho family of small GTPases into assembly of a dense actin network [3]. In addition to Arp2/3, numerous actin-binding proteins are required to maintain spatial regulation of the polymerization/depolymerization of actin filaments. For instance, capping proteins, such as Cap-ZIP, Lsp1 or the chaperone Hsp25 bind to the barbed ends and limit filament growth. In addition, recycling of actin monomers behind the leading edge is accomplished by the severing function of ADF/cofilin [4]. Directional migration is also controlled by the establishment of an intracellular gradient of PI(3,4,5)P3 (PIP3) and PI(3,4)P2 generated at the leading edge by Class I phosphoinositide 3-kinases (PI3Ks) [5]. Regulation of leading edge assembly and cell migration by factors downstream of small GTPases and PI3Ks is also accomplished by activation of numerous kinases, such as ROCK, PAK or LIM Kinase-1 (LIMK1) [6]. Activation of PAK has been shown to result in peripheral actin reorganization by phosphorylating substrates such as LIMK, which in turn phosphorylates and inactivates cofilin, a protein that promotes depolymerization of F-actin, leading to the stabilization of the actin filaments [7], [8]. Similarly, stress-dependent phosphorylation of capping proteins by MAPKAP-kinases (MKs) has been associated with regulation of the actin cytoskeleton [9].

Bone morphogenetic proteins (BMPs) belong to the transforming growth factor-β (TGF-β) superfamily. They have been shown to participate in the patterning and specification of several tissues and organs during vertebrate development and to regulate cell growth, apoptosis, differentiation and migration in different cell types [10]. BMP is also involved in cell migration. BMP-2 signaling is required for migration of neural crest pluripotent cells that generate craniofacial structures and the enteric nervous system [11], [12]. Furthermore, a number of studies indicated that BMPs mediate axon guidance and dendrite growth during neuronal development [13]. BMP-2 also induces in vitro migration of bone marrow mesenchymal progenitors, osteoblasts and endothelial cells [14]–[16].

Early events in canonical BMP signaling are initiated through the phosphorylation of specific receptor-regulated Smad proteins, namely R-Smad-1, -5 or -8. After phosphorylation, R-Smads form heteromeric complexes with the common mediator Smad-4. These Smad complexes migrate to the nucleus and activate the transcription of specific target genes [17]. In addition to Smads, BMPs activate other intracellular signaling pathways relevant to their cellular functions. Non-canonical BMP signaling includes Rho-like small GTPases, PI3K/Akt or various types of MAP kinases [18], [19]. Mechanistically, it is well established that TGF-β regulates TAK1/p38 pathway through recruitment and ubiquitylation of TRAF6 by activated receptor complexes [20], [21], however regulation of TAK1/p38 by BMP signaling is largely unknown. Although the signaling events leading to transcriptional activity induced by BMPs have been studied in depth, much less is known about the signaling pathways involved in BMP-2-mediated cell migration. Several studies indicated that BMP-mediated cell migration or axon and dendrite growth require activation of the small GTPase Cdc42, PI3-K as well as LIMK activities [22]–[26]. Most of these effects, at least for short term responses, are Smad independent but depend on LIMK binding to the long cytoplasmatic tail of BMPRII complexes [22]–[26]. In addition, previous data reported by our group indicated that activation of LIMK also requires the activation of PAKs through Cdc42 and PI3K [24].

Here we studied further the involvement of non-canonical Smad signaling in the induction of cell migration and the rearrangement of the actin cytoskeleton. We demonstrate by either pharmacological or genetic analysis that the BMP-2 effects on cell migration require the function of p38α and its downstream effector MK2. We also show that BMP-2 activation of p38/MK2 leads to phosphorylation of Hsp25 and that this phosphorylation and possibly other actin-capping proteins is relevant for BMP-2 induction of leading edge formation and cell migration. Altogether, the results presented here provide further support for the involvement of non-canonical BMP signaling mechanisms in actin reorganization and cell migration.

## Results

### p38 activity is required for cell migration

Activation of p38 MAPK has been shown to be relevant for the physiological effects of BMP-2 [27]. Furthermore, a direct mechanistic link between activated TGF-β receptor complexes and activation of p38 has been elucidated [20]–[21]. We investigated whether this direct involvement on p38 activation also applies for BMP-activated complexes. Treatment of C2C12 cells with BMP-2 increased the levels of endogenous active p38 after 20 min of ligand addition and peaked at 30–60 min (Fig. S1A). We also examinated whether this activation was a direct effect or depended on an autocrine secretion of a distinct cytokine. Incubation of cells with BMP-2 together with Noggin, which sequesters BMP-2 away from their receptors, abolished activation of p38. More importantly, treatment of C2C12 cells for 45 min with conditioned media of cells previously treated with BMP-2 for 30 min did not induce p38 activation in the presence of noggin in the conditioned media to block residual BMP-2 activity (Fig. S1B). These data suggest that p38 activation requires binding of BMP-2 to their signaling receptor complexes and does not rely on autocrine secretion of other cytokines.

**Figure 1:**
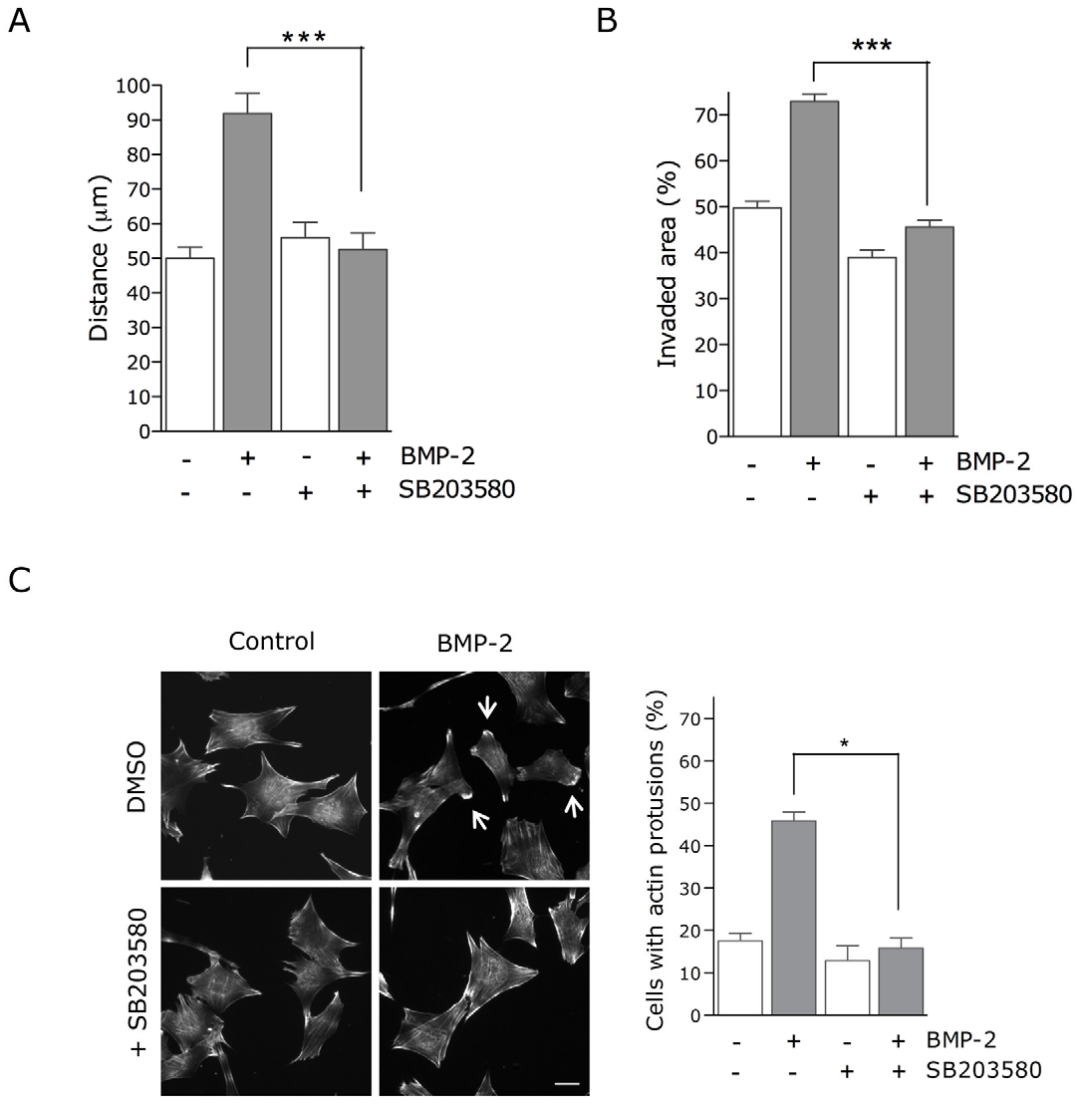
p38 activity is required for BMP-2-induced cell migration and actin reorganization in C2C12 cells. (**A**) Migration of C2C12 cells was analyzed by time-lapse video microscopy and cell-tracing in the presence or absence of BMP-2 and/or SB203580. Cells were imaged at 5-minute intervals for 16 h. Histogram shows migration tracks obtained from at least 80 cells in each experimental condition from two independent experiments. Values correspond to the mean ± SEM (*, p<0.0001, unpaired *t*-test). (**B**) Wounded C2C12 cell monolayers were allowed to migrate for 12 h in the presence or absence of BMP-2 and/or SB203580. A quantitative analysis of the invaded area was obtained from three photographed fields in at least three independent experiments. Values correspond to the mean ± SEM from three independent experiments (*, p<0,0001, unpaired *t*-test). (**C**) C2C12 cells were treated with SB203580 for 30 min, treated with 2 µM cytochalasin D (Cyto D) for 20 min and allowed to recover in the absence or presence of BMP-2 for 1 h. Actin was visualized with TRITC-conjugated phalloidin using a confocal microscope. Arrows indicate actin cortical protrusions. Bar, 20 µm. Cells with actin protrusions were quantified as shown in graph. Values correspond to the mean ± SEM from three independent experiments (*, p<0.05, paired *t*-test).

There is evidence that BMPs regulate cell migration and axon and dendrite growth during embryonic morphogenesis [11]–[12], [25]–[26], [28]. To test the role of p38 in BMP-2-induced cell migration, we performed an in vitro migration assay using time-lapse imaging and analyzing the trajectory of cells for 16 hours (Movies S1 and Movies S2). Quantification of cell migration revealed that in the presence of BMP-2 the distance covered by the cells almost doubled compared with the control (Fig. 1A). However, addition of SB203580 (a specific inhibitor of p38α/β activities) completely abolished the BMP-2-induced cell migration. To further confirm the role of p38 in the migration of C2C12 cells in response to BMP-2, wound-healing migration assays were performed. Cell monolayers were scrape-wounded and allowed to heal in the presence of BMP-2 and/or the p38 inhibitor. As shown in Figure 1B, in the presence of BMP-2 the wound was more efficiently invaded, whereas SB203580 completely abolished this positive effect of BMP-2.

Actin filament reorganization is likely to be involved in directed cell migration in all cell types and has been shown to be also involved in BMP-induced migration [26]. Therefore, we investigated whether p38 also played a role in the organization of F-actin in response to BMP-2. In agreement with previous data, BMP-2 induced the accumulation of cortical F-actin at the cell periphery [26]. The number of cells showing cortical actin protrusions more than doubled in the presence of BMP-2 (Fig. 1C). Pretreatment with p38 inhibitor completely abolished BMP-2-dependent induction of actin reorganization, suggesting that an intact p38 signaling pathway was required for the BMP-2 induced mobilization of the actin filament system.

### BMP-2-induced activation of the p38/MK2/Hsp25 signaling pathway

**Figure 2:**
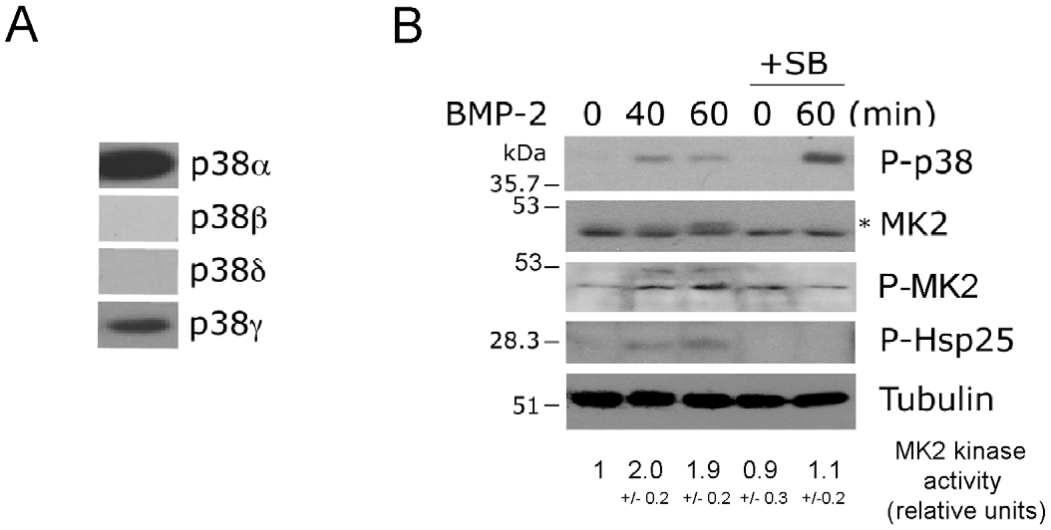
BMP-2 activates the MK2/Hsp25 pathway in a p38 dependent manner. (**A**) Immunoblot analysis of p38 proteins in C2C12 cell. (**B**) C2C12 cells were treated with BMP-2 and SB203580 as indicated, and cell lysates were analysed by immunoblotting with anti-phospho-p38, anti-MK2, anti-phospho-MK2, anti-phospho-Hsp27 and anti-Tubulin antibodies. The anti-MK2 antibody recognizes several MK2 forms; an asterisk indicates the phosphorylated form of MK2. C2C12 cells were treated with BMP-2 and SB2035380 as indicated, and endogenous MK2 was immunoprecipitated and subjected to an in vitro kinase assay with GST-Hsp25 as a substrate. Relative kinase activities are expressed as mean +/- S.E.M. of three independent experiments.

The p38 proteins are encoded by four different genes (p38 αβγδ) which display some substrate and inhibitor specificities [29]. To further characterize the BMP-2-mediated activation of p38, we first analyzed the expression pattern of the individual p38 proteins in C2C12 cells. Immunoblot analysis indicates that the major p38 proteins expressed were p38α and γ (Fig. 2A). RT-PCR analysis also demonstrated some level of expression of the β isoform [30]. p38 activity has been previously shown to mediate the activation of MK2/3 upon stimulation by stress signals or by several inflammatory cytokines [9], [31]. Similarly, MK2 downstream signaling has been shown to regulate cytoskeletal dynamics through phosphorylation of Hsp25/27 and other actin-capping proteins in several cell types [32]–[35]. We first confirmed the ability of BMP-2 to activate the MK2/Hsp25 pathway and the requirement of p38 for these effects. We examined the temporal profile of phosphorylation of MK2 and Hsp25, since phosphorylation of MK2 on Ser334 correlates with MK2 activation, and Ser 78 and 82 are the sites of Hsp25 phosphorylated by MK2 [36]. We demonstrated that BMP-2 induced MK2 phosphorylation and significantly (p<0.05) increased its kinase activity 40–60 minutes after BMP-2 addition. Increases in phosphorylation of MK2 also correlated with increased Hsp25 phosphorylation. These effects on MK2 phosphorylation and activity required activation of p38 since preincubation with the p38 inhibitor completely abolished the effects of BMP-2 on MK2 and Hsp25 (Fig. 2B).

**Figure 3:**
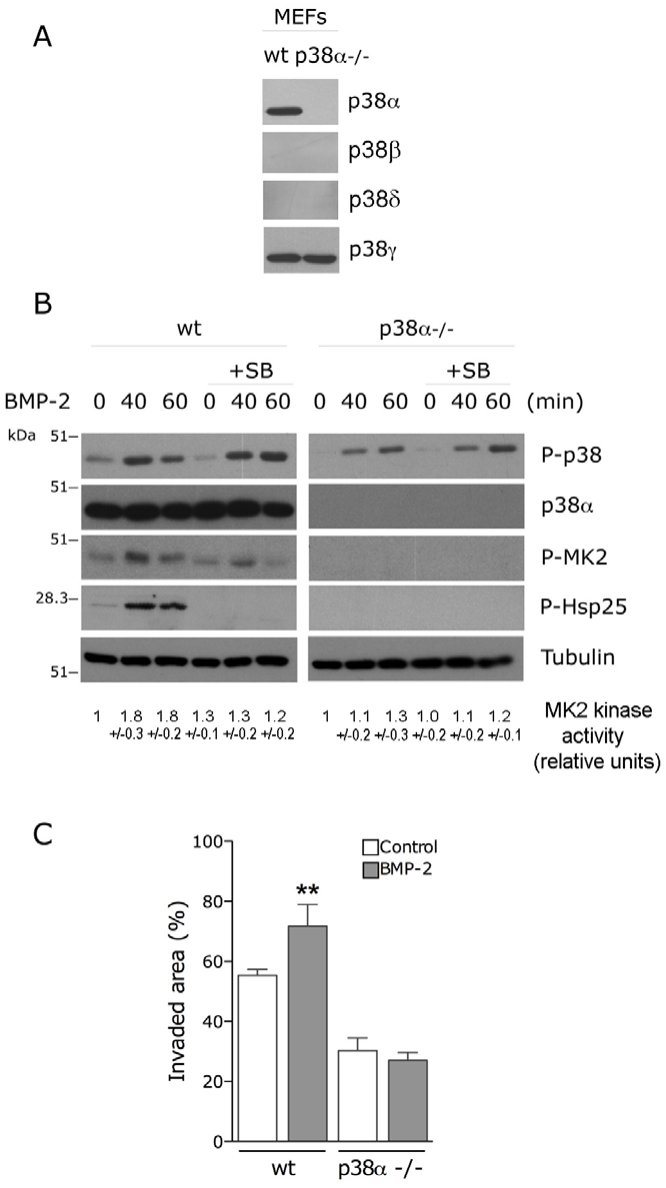
p38α activity is required for BMP-2-induced cell migration. (**A**) Immunoblot analysis of p38α, β, γ and δ isoform expression in p38α -/- MEF cells. (**B**) p38α -/- MEF cells were treated with BMP-2 and SB203580 as indicated and cell lysates were analysed by immunoblotting with anti-phospho-p38, anti-p38α, anti-phospho-MK2, anti-phospho-Hsp27 and anti-Tubulin antibodies. MK2 was also immunoprecipitated and subjected to an in vitro kinase assay with GST-Hsp25 as a substrate. Relative kinase activities are expressed as mean +/- S.E.M. of three independent experiments. (**C**) Wounded wt or p38α -/- MEF monolayers were allowed to migrate for 24 h in the presence or absence of BMP-2. Histogram shows the quantitative analysis of invaded area and values correspond to the mean ± SEM from three independent experiments (*, p<0.001 compared with the corresponding condition in absence of BMP-2, One-way ANOVA followed by Bonferroni's multiple comparison test).

There is some evidence to suggest that p38ãβ are the preferential activators of MK2/3 [31]. To further examine the requirement of distinct p38 proteins for migration, we analyzed the BMP2-induced migration of p38α -/- mouse embryonic fibroblasts (MEFs). We first confirmed that p38α -/- fibroblasts do not express the α isoform, showed no detectable expression of the β and δ isoforms measured by western blot, but express significant levels of the γ isoform (Fig. 3A). Similar to C2C12 cells, addition of BMP-2 to wt MEFs induced activation of the p38/MK2/Hsp25 signaling pathway which was completely abolished by preincubation with the specific p38α ~β inhibitor SB203580 (Fig. 3B). More importantly, addition of BMP-2 to p38α -/- MEFs was unable to induce activation of the downstream substrates MK2 or Hsp25. BMP-2 was still able to induce activation of other p38 proteins, visualized by immunoblot against phosphorylated p38 which recognizes all phosphorylated p38 proteins. However, this activation was not sufficient to transduce the signal to MK2 or Hsp25, further suggesting that the protein involved in this processes is p38α. We then examined the effect of p38α deletion on BMP-2-induced migration and showed that BMP-2 had no effect on the migration of the p38α -/- MEFs in wound-healing assays (Fig. 3C). p38α -/- MEFs showed a reduced basal migration levels compared with wt MEFs, data that has also been reported by other authors [37]. Altogether, these data suggest that p38α activity is required for BMP-2 signaling to MK2/Hsp25 and for BMP-2-induced actin reorganization and cell migration.

**Figure 4:**
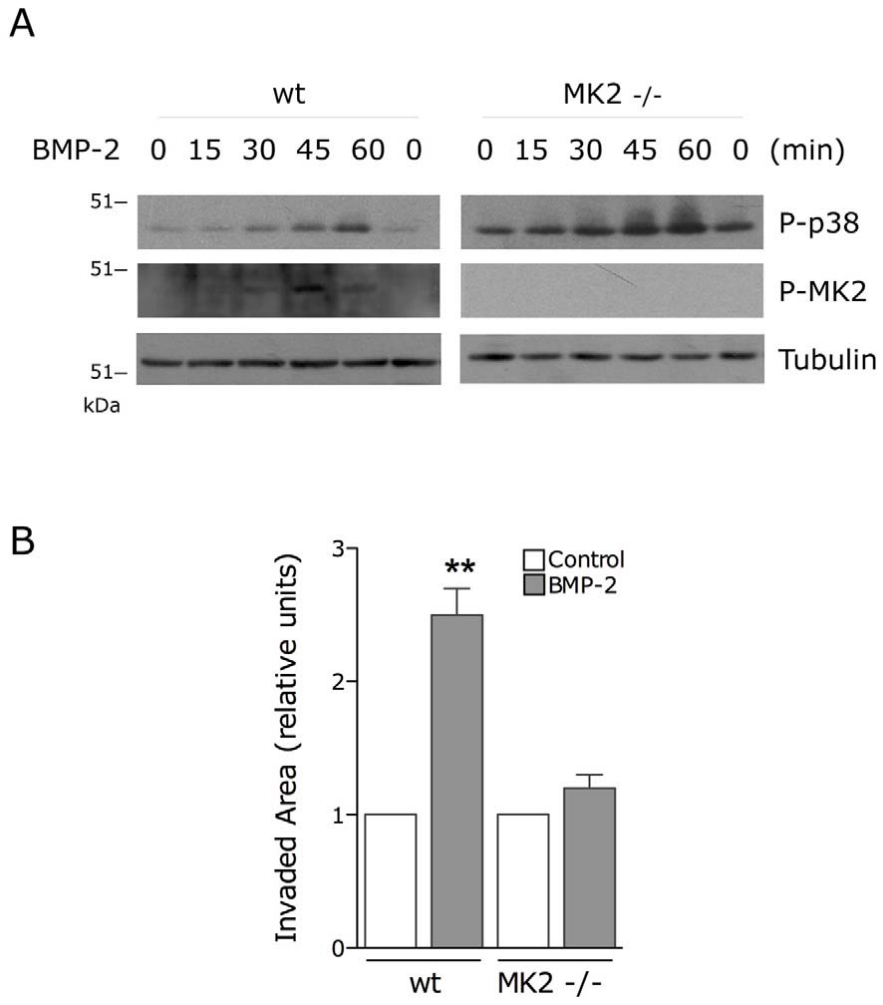
MK2 activity is required for BMP-2-induced cell migration. (**A**) wt and MK2 -/- MEF were stimulated with BMP-2 as indicated and cell lysates were analysed by immunoblotting with anti-phospho-p38, anti-phospho-MK2 and anti-Tubulin. (**B**) Wounded wt and MK2 -/- MEF monolayers were allowed to migrate for 24 h in the presence or absence of BMP-2. Histogram shows the quantitative analysis of invaded area and values correspond to the mean ± SEM from three independent experiments (*, p<0.001 compared with the corresponding condition in absence of BMP-2, One-way ANOVA followed by Bonferroni's multiple comparison test).

**Figure 5:**
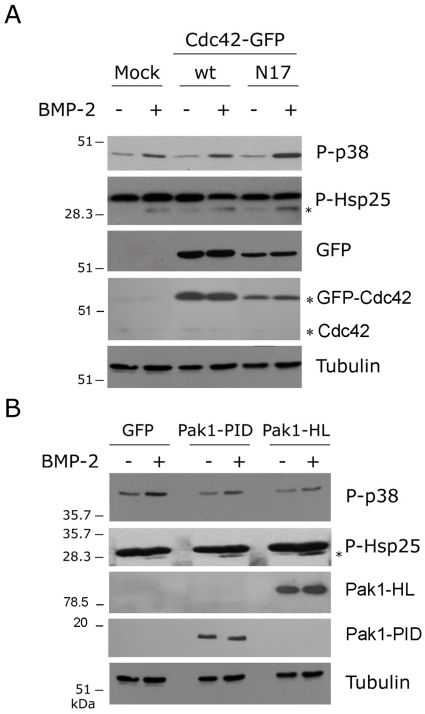
BMP-2 activates p38 independently of Cdc42/PI3K pathways. (**A**) C2C12 cells transfected with mock plasmid or GFP-tagged wild-type Cdc42 (GFP-Cdc42wt) or a dominant-negative form (GFP-Cdc42N17) were stimulated with BMP-2 and cell lysates were analyzed by immunoblotting with anti-phospho-p38 and anti-phospho-Hsp27 antibodies. The membranes were stripped and retested with anti-GFP, anti-Cdc42 and anti-Tubulin antibodies. Bands corresponding to GFP-Cdc42 overexpressed forms and endogenous Cdc42 are indicated by asterisks. (**B**) C2C12 cells transfected with GFP or myc-tagged plasmids for a constitutive active form of PAK1 (Pak1-HL) or a dominant-negative form (Pak1-PID) were stimulated with BMP-2 and cell lysates were analyzed by immunoblotting with anti-phospho-p38 and anti-phospho-Hsp27 antibodies and reprobed with anti-myc or anti-Tubulin antibodies. The anti-phospho-Hsp27 antibody recognizes several unspecific bands, but only the one indicated with an asterisk corresponds to the phosphorylated form of Hsp25.

To determine whether MK2 is required for the BMP-2 effects on migration downstream of p38α we analyzed the BMP2-induced migration of MK2 -/- MEFs. MK2 -/- fibroblasts showed enhanced p38 phosphorylation levels compared with wt MEFs, coherent with data from other authors indicating that MKP-1 p38 phosphatase expression is regulated by p38 pathway downstream of MK2 (Fig. 4A)[38]. Similar to p38α-/- MEFs, BMP-2 had no significant effect on the migration of MK2-/- MEFs in wound-healing assays (Fig. 4B), suggesting that p38 controls actin polymerization by a mechanism dependent on activation of MK2. We have previously shown that BMP-2-induced reorganization of the actin cytoskeleton and cell migration requires signals emanating from Cdc42 and PI3K which converge on activation of PAK [24]. Similarly, some reports suggested that Cdc42 function could be either upstream or downstream of p38 upon TGF-β activation of PC-3U cells [39]–[40]. We therefore determined whether the BMP-2-activation of p38/MK2/Hsp25 depends on Cdc42 activity. C2C12 cells were transfected with either wild type Cdc42 or the dominant-negative mutant Cdc42-N17 and the phosphorylation of p38 and Hsp25 was analyzed after BMP-2 stimulation. Cells transfected with dominant-negative form of Cdc42 showed similar activation of p38 as well as Hsp25 to that observed in cells transfected with GFP alone or wild type Cdc42 (Fig. 5A). Also, the expression of a constitutive active form of PAK1 (PAK-H83,86L) or a dominant-negative form (PAK-PID) did not affect the activation of either p38 or Hsp25 (Fig. 5B). These observations suggest that BMP-2 activated the p38 and the Cdc42/PI3K pathways independently and that both signaling pathways are required for the BMP-2-induced cell migration.

**Figure 6:**
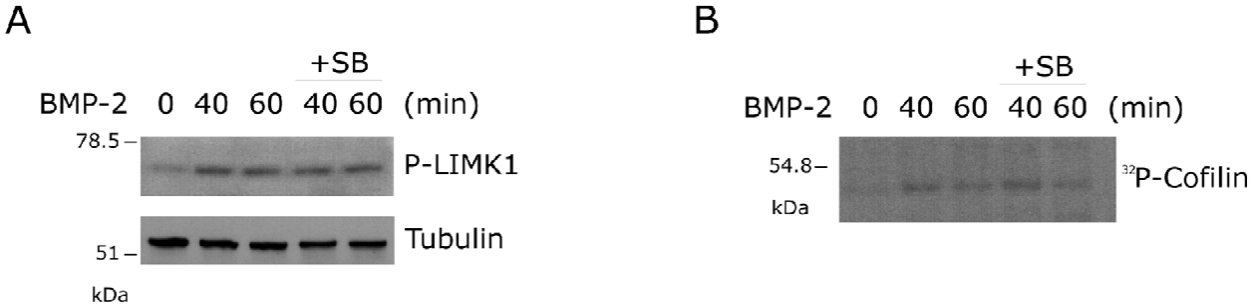
BMP-2 stimulation of LIMK1 activity is independent of p38 pathway. (**A**) C2C12 cells were treated with BMP-2 and SB203580 as indicated and cell lysates were analysed by immunoblotting with anti-phospho-LIMK1 and anti-Tubulin antibodies. (**B**) C2C12 cells were treated with BMP-2 and SB2035380 as indicated and endogenous LIMK1 was immunoprecipitated and subjected to an in vitro kinase assay with GST-cofilin as a substrate.

Previous reports indicate a direct interaction of LIMK1 with the long cytoplasmic tail of the BMP receptor type II [22]–[23]. Although these studies reached different conclusions regarding the mechanism by which BMP increases LIMK1 activity, it seems clear that BMP signaling in dendritogenesis and synaptic stability requires LIMK1 activity downstream of BMP receptors [23]–[25]. Moreover, it was reported that MK2 activation of LIMK1 through phosphorylation of Ser323 is required in VEGF-induced actin remodeling and cell migration [41]. We therefore tested whether LIMK1 activity was regulated by p38/MK2 signaling in response to BMP-2. As shown in Figure 6A, BMP-2 enhanced LIMK1 phosphorylation at its activation loop (Thr508) with maximal effect at 40–60 min and inhibition of p38 did not significantly block this phosphorylation. We also examined if p38/MK2 regulated LIMK activity through mechanisms other than PAK phosphorylation of Thr508. Because LIMK1 directly phosphorylates cofilin, we analysed whether the BMP-2-dependent LIMK1 phosphorylation correlated with an increase in LIMK1 activity measured by phosphorylation of recombinant cofilin. BMP-2 stimulation for 40–60 min resulted in increased LIMK1 mediated cofilin phosphorylation (Fig. 6B). The effect of BMP-2 on LIMK1 activity was only slightly decreased in the presence of SB203580 inhibitor (Fig. 6B). These observations indicate that BMP-2 stimulation of LIMK1 activity mainly occurs through p38-independent mechanisms.

### Hsp25 phosphorylation is required for BMP-2 induced cell migration and localizes in cortical actin protrusions

**Figure 7:**
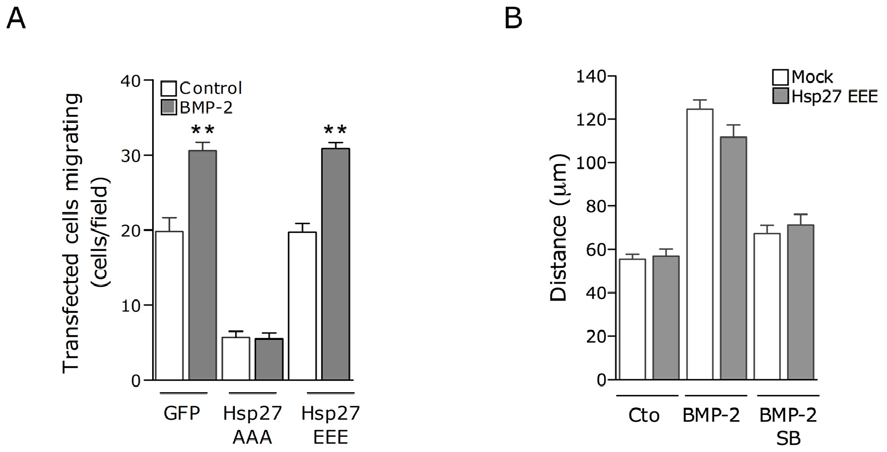
Hsp25 is required for BMP-2-induced cell migration. (**A**) C2C12 cells transfected with GFP or the GFP-tagged Hsp27 phospho-deficient mutant (Hsp27AAA) or a phospho-mimetic mutant (Hsp27EEE) were analyzed in a wound-healing migration assay performed for 12 h in the presence or absence of BMP-2. Means ± SEM of three independent experiments (*, p<0.001 compared with the corresponding situation in absence of BMP-2, One way ANOVA followed by Bonferroni's Multiple Comparison Test). (**B**) C2C12 cells transfected with GFP or GFP-tagged Hsp27EEE were analyzed by time-lapse video microscopy in the presence of BMP-2 and SB203580 as indicated. Cells were imaged at 5-minute intervals for 16 h. Histogram shows migration tracks obtained from at least 80 cells in each experimental condition from two independent experiments. Values correspond to the mean ± SEM.

A prominent in vivo substrate of MK2 shown to have a role in cell migration is Hsp25 (Hsp27 for the human gene). Hsp25 acts as an actin-capping protein and its phosphorylation is necessary for the formation of F-actin and increasing the rate and extent of actin polymerization in lamellipodia [36]. To examine whether phosphorylation of Hsp25/27 is required in BMP-2-induced migration, we co-expressed in C2C12 cells GFP as well as an Hsp27 phosphorylation mutant, where all three serines phosphorylated by MK2 were mutated to alanine, and a phosphorylation mimicking mutant, where all serines were mutated to aspartic acid. We then performed the wound-healing migration analysis of these cells visualizing the transfected ones by detecting the GFP signal. GFP-transfection alone did not significantly affect the ability of the cells to repopulate the wound after stimulation with BMP-2 (Fig. 7A). However, expression of the phosphorylation deficient mutant resulted in decreased migration and completely abrogated the ability of BMP-2 to stimulate the migration of the transfected cells, while the phospho-mimicking mutant had no effects on basal or stimulated migration (Fig. 7A). Thus suggesting that phosphorylation of Hsp25 is required for the migration of C2C12 cells in response to BMP-2. We also analyzed the ability of the phospho-mimetic mutant of Hsp27 to affect the p38/MK2 signaling by in vitro migration assay using time-lapse imaging and analyzing the trajectory of cells transfected with Hsp27-EEE for 16 hours. Surprisingly, addition of SB203580 completely abolished the BMP-2 induced cell migration irrespective of the expression of the phospho-mimetic mutant (Fig. 7B). These data suggest that either the mutant was unable to compensate fully for endogenous phosphorylation or alternatively, that other proteins regulated by p38 might play a role in these migratory effects.

**Figure 8:**
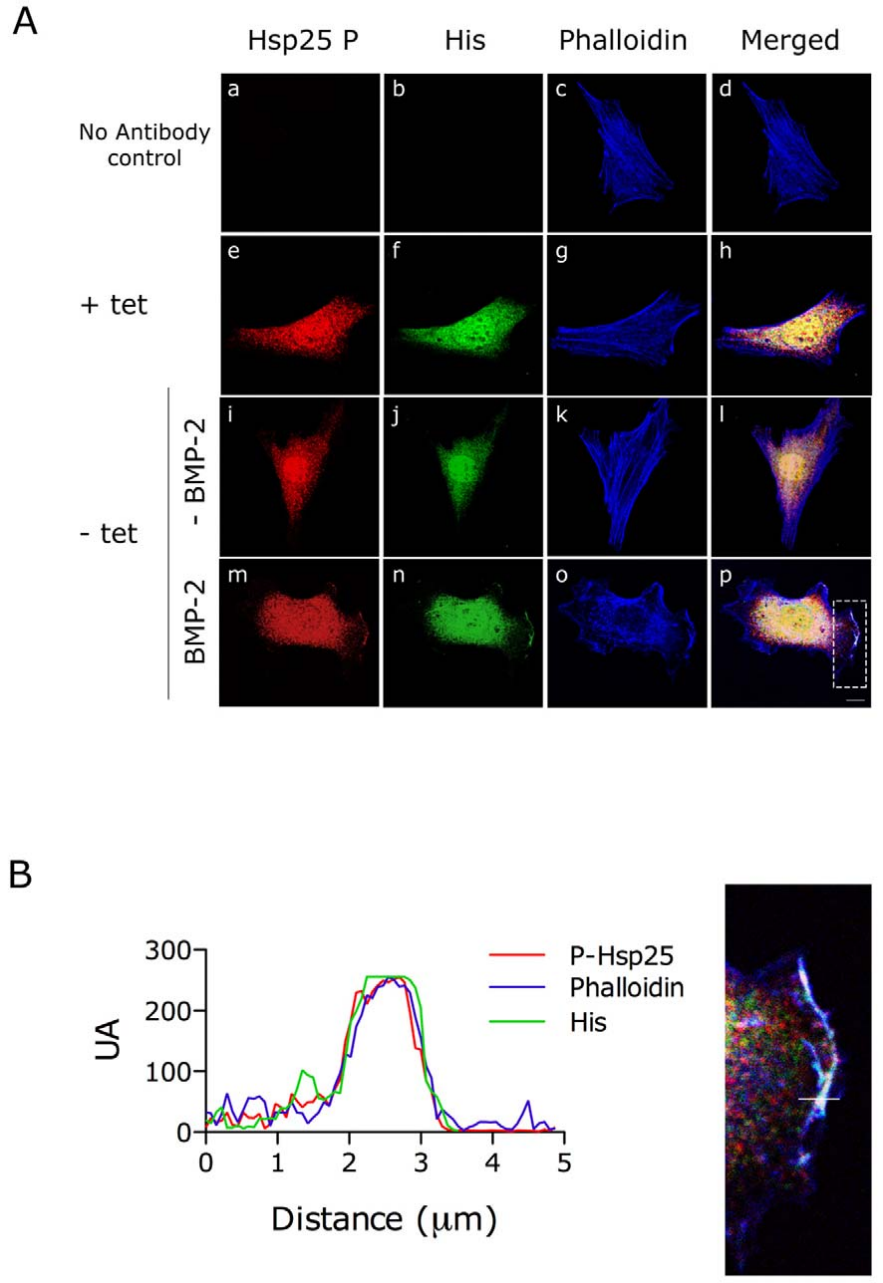
Phosphorylated Hsp25 and BMPRII colocalize in membrane protusions upon BMP-2 stimulation. (**A**) Immunostaining of phospho-Hsp25 (red) and His (green) of C2C12 cells overexpressing His-tagged BMPRII under a tetracycline (Tet-off) regulated promoter. Cells were serum-starved for 16 h, pre-treated with cytochalasin D and allowed to recover for 1 h in the absence (i-l) or presence (m-p) of BMP-2. Actin cytoskeleton was stained with Alexa Fluor 633 phalloidin (blue) and merged images are shown. (a-d), Controls where primary antibodies have been omitted from the incubation. (e-h), Immunostaining of the His-tagged BMPRII overexpressing clone incubated in the presence of 30 ng/ml of tetracycline for 24 h showing the diffuse background nuclear staining by anti-His antibody. Scale bar, 40 µm. (**B**) Spatial quantification was performed along a path across the plasma membrane, indicated by a white line in the detail amplification of the merged image above. Red, green and blue fluorescence was quantified separately and plotted as a function of distance along the path.

Unphosphorylated Hsp25 is preferentially localized in the perinuclear region of the cytoplasm, whereas both the phosphorylated and unphosphorylated proteins are present at the lamellipodia after stimulation [33]. We analyzed the localization of the phosphorylated Hsp25 and BMPRII on C2C12 cells stably expressing His-tagged BMPRII under a tetracycline-off responsive promoter [26]. The images in Figure 8 show that tetracycline treated cells present diffuse background nuclear staining with anti-His antibody. In untreated cells, staining for phosphorylated Hsp25 showed a diffuse perinuclear cytoplasmatic staining. Twenty four hours after tetracycline removal and after BMP-2 addition for 60 min, co-staining showed that, in addition to the intense perinuclear labeling, phosphorylated Hsp25 colocalized with overexpressed BMPRII and cortical actin in membrane protrusions (Fig. 8A lower panel, Fig. 8B). These data suggest that, upon BMP-2 stimulation, phosphorylated Hsp25 colocalizes with BMP receptor complexes in cortical actin protrusions and that, acting as a downstream effector of p38/MK2, it may play a critical role in BMP-2-induced cell migration.

## Discussion

MAPK signaling has been shown to be required for BMP physiological effects such as developmental patterning and organogenesis or apoptosis and is involved in pathological events such as aberrant epithelial-mesenchymal transition, tumor progression and invasion [19], [27], [42]–[43]. Depending on the cell type and condition studied, the effects of BMP on MAPK activities differ in kinetics and magnitude. In some cases, MAPK activities, and their downstream effects, are induced very rapidly suggesting a non-transcriptional mechanism, whereas in other conditions activation requires more time suggesting an indirect mechanism. A mechanism for rapid activation of the p38 pathway by TGF-β has been elucidated which involves recruitment and ubiquitylation of TRAF6/TAK1 by activated receptor complexes [20]–[21]. Here, we report that BMP-2 also stimulates p38 directly downstream of activated BMP receptor complexes and that stimulation of p38 is required for actin cytoskeletal reorganization and cell migration. In addition, our results show clearly that p38α is a major protein responsible for the migratory effects of BMP-2 in mesenchymal cells.

Previous studies have suggested that, in response to TGF-β, p38 plays a role in the control of cell migration [44]–[45]. Furthermore, studies in Drosophila indicate that p38 is required downstream of *Dpp*, the Drosophila homolog of BMPs, during wing morphogenesis [46]. Our results demonstrate, for the first time, that the p38/MK2/Hsp25 signaling pathway is important for BMP-2-induced accumulation of actin in cortical protrusions and migration of mesenchymal cells. We base this conclusion on the following observations. First, BMP-2 activates p38/MK2/Hsp25 signaling with the same temporal profile of induction of actin reorganization [24]. Second, BMP-2-induced formation of actin protrusions and cell migration is completely abolished by the specific p38α/β inhibitor, SB203580. Third, MEFs from mice deficient in p38α or MK2 are refractory to the stimulatory effects of BMP-2 on migration. Finally, overexpression of a phosphorylation resistant mutant of Hsp27 also blocked BMP-2-induced migration. These results are consistent with data showing that mice deficient in p38α have defects in angiogenesis and that p38α -/- MEFs display impaired migration, either basal or in response to chemotactic factors [37], [47]. Other groups also demonstrate that directional migration in response to several growth factors as well as physiological conditions is abolished in MK2 deficient MEFs [37], [48]–[49]. Moreover, whereas MK2 activity is abolished in the p38α deficient mice, mice lacking p38β are healthy and exhibit normal MK2 activation further suggesting that p38α is the predominant protein mediating MK2 effects on migration [47], [50]. MK2 and inactive p38α form a stable complex in the nucleus [51]. Upon activation, p38α phosphorylates MK2 at the regulatory sites, a masked nuclear export sequence becomes exposed and the active complex is translocated to the cytoplasm [52]. Once in the cytoplasm, the active p38/MK2 complex is able to localize at the leading edge of migrating cells and regulate chemotactic cell movements [34].

A growing body of evidence has identified Hsp25/27 as the substrate that mediates most of the MK2 effects on cell chemotaxis [32], [34], [53]–[54]. Phosphorylation of Hsp25/27 by MK2 has been associated with modification of its oligomerization and regulation of the actin cytoskeleton [9]. Phosphorylation of Hsp25/27 eliminates its cap-binding activity on barbed ends therefore allowing actin polymerization and remodeling to take place concomitantly with the binding of additional proteins to actin [55]. Our results suggest that this may also be the case for BMP-2 induced migration. Hsp25/27, phosphorylated in response to BMP-2, localizes in actin protrusions together with BMPRII and the expression of the phosphorylation resistant mutant of Hsp27 was able to completely block migration in scratch-wound assays. However, migration of cells expressing a phospho-mimetic form of Hsp27 is also stimulated by BMP-2 and the p38α/β inhibitor is still able to block the BMP-2 migration effects on these cells. This opens the possibility of a more complex regulation of cytoskeletal architecture by BMPs. Other MK2 substrates include additional cap-binding proteins such as Lsp1, Cap-ZIP and the p16 subunit-Arc of the Arp2/3 complex which are also involved in actin remodeling and could contribute to the BMP-2 effects [56]–[58]. Moreover, previous data from our group demonstrated that Cdc42 and PI3K, acting in parallel but independently, are also required for the BMP-induced cytoskeletal effects through activation PAK and LIMK resulting in cofilin phosphorylation and increased actin polymerization [24]. Thus, it is possible that BMP-2 activation of separate, non-redundant pathways converges in the spatial regulation of actin cytoskeleton by BMPs through regulation of distinct proteins involved in actin polymerization.

Our analysis also included the possibility of cross-talk between the p38α/MK2 and PAK/LIMK signaling downstream of the BMP receptors. Reports suggest that phosphorylated Hsp25/27 could regulate Akt activity through scaffolding of MK2 to Akt signal complex in leukocytes [59]. In addition, Kobayashi et al. demonstrated that, in endothelial cells stimulated with VEGF, LIMK1 activity is directly regulated by its phosphorylation by MK2, and suggested that the VEGF effects were independent of LIMK1 phosphorylation by PAK on Thr508 [41]. In agreement with previous results, our data indicate that, in C2C12 cells, BMP-2 activates LIMK1 phosphorylation on Thr508, resulting in cofilin phosphorylation. We also demonstrated that BMP-induced LIMK1 phosphorylation on Thr508 and activity is mostly independent of p38/MK2 activity using the specific p38 inhibitor. Moreover, overexpression of constitutive active MKK6 did not modify LIMK activity towards cofilin (data not shown). Previous reports indicated a direct interaction and activation of LIMK1 via the long cytoplasmic tail of the BMP receptor type II [22]–[23]. Thus, since BMPRII, and associated LIMK, as well as MK2/Hsp25 colocalized in actin protrusions we cannot dismiss the possibility that phosphorylation of LIMK by MK2 may be involved in anchoring LIMK1 to specific locations and might allow the confinement of LIMK1 activity and cofilin phosphorylation.

**Figure 9:**
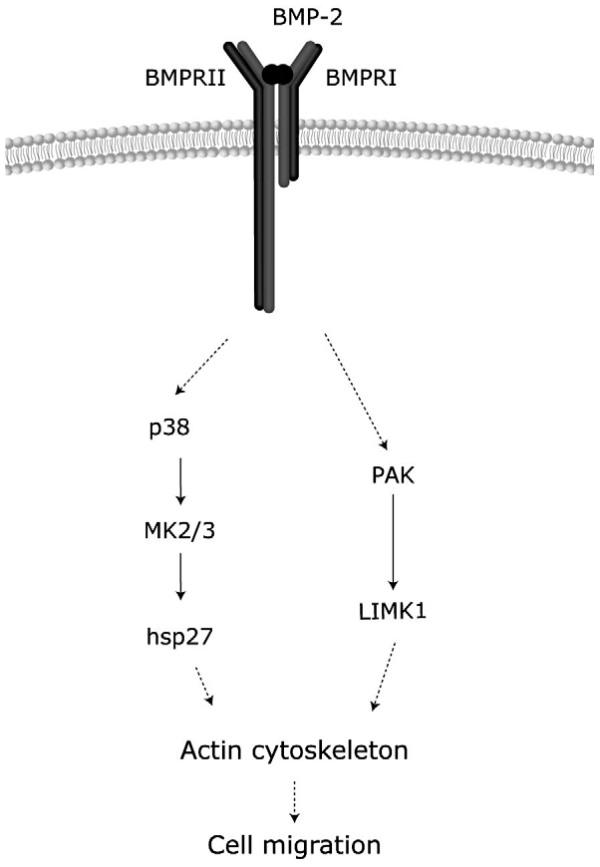
Model of the mechanism of regulation of cell migration by BMP-2. Activation of BMP receptor transduces the signal to changes in actin cytoskeleton through parallel activation of the p38-MK2-Hsp25 and the PAK-LIMK-cofilin pathways.

Previous studies also indicated that, in some instances, small GTPases of the Rho family or PAK activities are involved in activation of p38. For example, βPix, through its interaction with PAK and acting as a GEF for Cdc42/Rac, regulates MKK3/6 and p38 activities and both pathways contributed to the actin filament remodeling induced by PDGF in fibroblasts [60]. Similarly, p38 is activated by constitutive active forms of Cdc42/Rac or inhibited by dominant negative forms of Cdc42 or PAK1 in endothelial or fibroblastic cells under different stimuli [61]–[63]. However, we also demonstrate that activation of the p38/MK2/Hsp25 pathway by BMP-2 is independent of the activities of either Cdc42 or PAK1. Thus, our results indicate that, although Cdc42/PAK/LIMK and p38/MK2/Hsp25 pathways are both required for the BMP-2 migratory effects, BMP-2 is able to activate both routes mostly independently since the blockage of one pathway does not alter its ability to stimulate the other one (Fig. 9). Altogether, our data suggest that multiple signaling pathways, acting in parallel and modulating specific actin regulatory proteins, play a role in integrating responses during BMP-induced actin reorganization and cell migration.

## Materials and Methods

### Cell culture, plasmids and reagents

Vectors encoding Cdc42wt-GFP and Cdc42-T17N-GFP were kindly provided by X. Bustelo (CSIC, Salamanca, Spain). Vectors encoding PAK1-PID (aa 83-149) and PAK1-HL (H83,86L) were provided by G. Bokoch (The Scripps Research Institute, La Jolla, CA). Vectors encoding Hsp27-AAA (S15,78,82A) and Hsp27-EEE (S15,78,82E) were provided by K. Kim (Seoul National University, Korea). BMP-2 was a generous gift from Wyeth (Cambridge, MA). In all the experiments BMP-2 was used at a final concentration of 3 nM, and before BMP-2 stimulation cells were serum-starved for 16 h. Noggin was from R&D. The inhibitor SB203580 (Calbiochem) was added to the media 30 min before stimulation of cells with BMP-2 and used at a final concentration of 10 µM. Purified cofilin 1 was from Upstate Biotechnology (Lake Placid, NY). Antibodies used were Tubulin (Sigma); p38β (Zymed); p38γ (Upstate); GFP (Roche), His (Amersham); p38α, p38δ, Phospho-p38 MAP Kinase (Thr180/Tyr182), MAPKAP-2, Phospho-MAPKAP-2 (Thr222), Phospho-Hsp27 (Ser15), LIMK1 and Phospho-LIMK1 (Thr508)/LIMK2(Thr505) (Cell Signaling, Beverly, MA). C2C12 cells with inducible expression of His-tagged BMPRII have been described previously [24]. C2C12 cell line and mouse embryonic fibroblasts (MEF) wild type, p38α -/- (a gift from A. Nebreda, CNIO, Spain) and MK2 -/- (gift from M. Gaestel, Hannover Medical School, Germany) were maintained in DMEM supplemented with 10% FBS, antibiotics and glutamine. Cells were transfected using Lipofectamine LTX (Invitrogen, Carlsbad, CA).

### Immunoblotting, immunoprecipitation and protein kinase assays

Protein extracts were subjected to SDS-PAGE and immunoblotted as previously described [64]–[65] or used for in vitro kinase assay.

For kinase assays, cells were grown to confluency, starved in serum-free medium for 16 h and stimulated with 3 nM BMP-2 for the times indicated. Cells were washed twice in cold PBS and lysed on ice with 500 µl per 10 cm dish of ice-cold lysis buffer [40 mM Tris-HCl (pH 7.5), 150 mM NaCl, 0.2% NP-40, 10% glycerol, 50 mM NaF, 40 mM β-glycerophosphate, 200 µM, Na_3_VO_4,_ 100 µM phenylmethylsulfonyl fluoride, 1 µM pepstatin A, 1 µg/ml leupeptin, 4 µg/ml aprotinin]. Lysates were pre-cleared by centrifugation at 10000 rpm for 10 min at 4°C and equivalent protein amount (500-700 µg) was incubated overnight at 4°C with anti LIMK1 or anti MAPKAP-2 polyclonal antibodies. Immune complexes were collected with protein A-Sepharose and protein G-Sepharose (GE Healthcare) and washed four times in lysis buffer and twice in kinase buffer [50 mM HEPES pH 7.4, 150 mM NaCl, 1 mM MgCl_2_, 10 mM NaF, 1 mM Na_3_VO_4_, 5% glycerol, 1 mM dithiothreitol, 1 mM phenylmethylsulfonyl fluoride for LIMK activity or 50 mM β-glycerophosphate, 0.1 mM EDTA, 4 mM magnesium acetate] prior to incubation in 50 µl of the respective kinase buffers containing 5 µM ATP, 5 µCi of [γ-^32^P] ATP per reaction, and 5 µg of GST-cofilin for LIMK or 5 µg Hsp25 for MK2. After 20 min at 30°C, reactions were stopped by addition of SDS sample buffer and boiled for 10 min. The reaction mixture was separated by SDS-PAGE and analysed by autoradiography.

### F-actin staining and immunofluorescence

Cells were grown on glass coverslips in 12-well plates, starved in serum-free medium for 16 h and incubated with 2 µM cytochalasin D (Sigma) for 20 min, washed 5 times with media and allowed to recover in the presence or absence of 3 nM BMP-2. As described previously [24], Cytochalasin D was used to transiently disrupt the actin cytoskeleton in order to emphasize the BMP-2-dependent effect. Cells were fixed in 4% paraformaldehyde in PBS for 20 min at room temperature, washed twice in PBS and permeabilized for 4 min in PBS containing 0.2% Triton X-100, and then blocked in TBS containing 2% BSA for 45 min. To visualize F-actin, cells were incubated with 1 µM TRITC-conjugated phalloidin (Sigma) and washed three times with PBS before mounting onto slides. For the immunofluorecence assay, cells were stained with anti-phospho-Hsp27 and anti-His antibodies at 1∶50 dilution followed by goat anti-mouse IgG conjugated Alexa 488 and goat anti-rabbit IgG conjugated Alexa 555 at 1∶400. Images were acquired using LEICA TCS-SL NIKON-E800 confocal microscope linked to a Diagnostic Instruments Inc. model SPOT-JR camera.

### Time-lapse video microscopy

C2C12 cells were grown on 4-well coverslip-bottom plates, serum-starved for 16 h and stimulated with 3 nM BMP-2 as indicated, before being placed at 37°C in a CO_2_ incubation system. Time-lapse images (typically 4 Z-stack sections) were recorded at 5-min intervals for 16 h using a LEICA TCS-SL NIKON-E800 confocal microscope linked to a Diagnostic Instruments Inc. model SPOT-JR camera. Individual cells were traced from the resulting time-lapse movies and analysed using LCS Analysis Software. Cell migration was analysed by marking the position of the nucleus in individual cells in each frame to obtain migration tracks. Some pictures were made as movies (Movies S1, Movies S2).

### Wound-healing migration assay

C2C12 or MEFs cells were grown to confluence and serum-starved for a minimum of 16 h to establish quiescence. Cell monolayers were wounded with a plastic tip and washed with media to remove detached cells. The wound was allowed to close in the presence or absence of 3 nM BMP-2. The wound was photographed in a phase-contrast microscope (LEICA DM IRB microscope linked to an OLYMPUS DP50 camera) and the rate of cell migration was measured as the percentage of the invaded area with respect to the initial wound area.

### Statistical analysis

Statistical analysis was performed using Student's *t*-test or One-way ANOVA followed by Bonferroni's multiple comparison tests. Quantitative data are presented as means ± SEM. Differences were considered significant at p values <0.05: ^*^p<0.05, ^**^p<0.001, and ^***^p<0.0001.

## Supporting Information

Figure S1 **BMP-2 induces direct activation of p38.** **(A)** C2C12 cells were stimulated with BMP-2 and cell lysates analyzed by immunoblotting with anti-phospho-p38 and anti-tubulin antibodies. **(B)** C2C12 cells were stimulated with the cytokines indicated. For conditioned media assays, cells were treated with BMP-2 and after 30 min the media was collected, incubated with 0.6 µg/ml Noggin, for 5 min and used to stimulate serum-starved C2C12 for 45 min.
(TIF)

Movie S1 **Basal migration of C2C12 cells.** C2C12 cells were grown on 4-well coverslip-bottom plates and serum-starved for 16 h. Time-lapse images (typically 4 Z-stacks sections) were recorded at 5-min intervals for 16 h using a LEICA TCS-SL NIKON-E800 confocal microscope linked to a Diagnostic Instruments Inc. model SPOT-JR camera. Cells were maintained at 37°C in a CO_2_ incubation system.
(Mp4)

Movie S2 **BMP-2 stimulates migration of C2C12 cells.** C2C12 cells were grown on 4-well coverslip-bottom plates, serum-starved for 16 h and stimulated with 3 nM BMP-2. Time-lapse images (typically 4 Z-stacks sections) were recorded at 5-min intervals for 16 h using a LEICA TCS-SL NIKON-E800 confocal microscope linked to a Diagnostic Instruments Inc. model SPOT-JR camera. Cells were maintained at 37°C in a CO_2_ incubation system.
(Mp4)
